# Knowledge of Cervical Cancer Screening and Prevention by Human Papillomavirus Deoxyribonucleic Acid and Human Papillomavirus Vaccination among Women Attending a Tertiary Care Centre

**DOI:** 10.31729/jnma.8248

**Published:** 2023-08-31

**Authors:** Sapana Amatya Vaidya, Lisa Roka Magar, Silpina Budha Magar

**Affiliations:** 1 Department of Gynecology and Obstetrics, Paropakar Maternity and Women's Hospital, Thapathali, Kathmandu, Nepal; 2 Civil Service Hospital, Minbhawan, Kathmandu, Nepal; 3 Pokhara Academy of Health Sciences, Western Regional Hospital, Pokhara, Kaski, Nepal

**Keywords:** *cervical cancer*, *human papillomavirus*, *pap smear*, *sexual intercourse*, *vaccination*

## Abstract

**Introduction:**

Cervical cancer is one of the leading causes of morbidity and mortality among women globally as well as in Nepal. It is attributable to persistent infection by high-risk human papillomavirus, especially human papillomavirus-16 and human papillomavirus-18. The aim of this study was to find out the knowledge of cervical cancer screening and prevention by human papillomavirus deoxyribonucleic acid and human papillomavirus vaccination among women attending a tertiary care centre.

**Methods:**

A descriptive cross-sectional study was conducted in patients attending the outpatient Department of Gynaecology in a tertiary care centre from 18 March to 30 April 2023. After calculating sample size and taking a convenience sampling a survey questionnaire on knowledge of Cervical Cancer Screening and Prevention by Human Papillomavirus Deoxyribonucleic Acid and Human Papillomavirus Vaccination was collected. The point estimate was calculated at a 95% confidence interval.

**Results:**

Among 508 women, 42 (8.25%) (5.86-10.64, 95% Confidence Interval) had knowledge of cervical cancer screening and prevention by human papillomavirus deoxyribonucleic acid and human papillomavirus vaccination. According to the questionnaires with a total sample of 508, 164 (32.28%) know about cervical cancer, 15 (2.95%) know about HPV infection, 14 (2.76%) know about HPV infection causes cervical cancer, and 21 (4.13%) know about HPV transmitted through multiple sex partners.

**Conclusions:**

The knowledge of cervical cancer screening and prevention by human papillomavirus deoxyribonucleic acid and human papillomavirus vaccination among women is very low. This study recommends having a health education and awareness programme on it to increase knowledge.

## INTRODUCTION

Cervical cancer, caused by human papillomavirus (HPV) especially HPV-16 and HPV-18 is one of the leading causes of morbidity and mortality among women globally as well as in Nepal.^1,2^ About 11.4 million women population of aged 15 years and older in Nepal are at risk of developing cervical cancer.^3^ In 2020, an estimated 604 000 women were diagnosed with cervical cancer worldwide and about 3,42,000 women died from the disease.^4^

The World Health Organization's (WHO) global strategy includes vaccinating 90% of eligible girls against HPV by 2030; screening 70% of eligible women at least twice in their lifetimes and effectively treating 90% of those with a positive screening test/a cervical lesion, including palliative care when needed.^4^

The aim of this study was to find out the prevalence of knowledge of cervical cancer screening and prevention by human papillomavirus deoxyribonucleic acid and human papillomavirus vaccination among women attending a tertiary care centre.

## METHODS

This descriptive cross-sectional study was conducted in the Outpatient Department of Gynecology at Paropakaar Maternity and Women's Hospital, Thapathali, Kathmandu, Nepal from 18 March 2023 to 30 April 2023. An ethical approval was taken from the Institutional Review Board (IRB) (Reference number: 780/2079/80). The women in the age group 30-65 years were included in the study. The women with incomplete hospital records were excluded. An 11 points questionnaire on Knowledge of Cervical Cancer Screening and Prevention by Human Papillomavirus Deoxyribonucleic Acid and Human Papillomavirus Vaccinations was distributed. The patients were asked to return the self-reported questionnaires using convenience sampling methods. The sample size was calculated by using the following formula:


$$\begin{array}{ll} \text{n} & ={\text{Z}}^{2}\times \frac{\text{p}\times \text{q}}{{\text{e}}^{\text{2}}}\\ & =1.96^{2}\times \frac{0.50\times 0.50}{0.05^{2}}\\ & =385 \end{array}$$

Where,

n = minimum required sample size
Z = 1.96 at 95% Confidence Interval (CI)
p = prevalence taken as 50% for maximum sample size calculation
q = 1-p
e = margin of error, 5%

The minimum required sample size calculated is 385. However, we took 508 samples.

Patient demographics; age, education status, and profession were collected with the survey questionnaires. The questionnaires included selfreported questionnaires to state yes or no on cervical cancer, HPV, HPV causing cervical cancer, that having sexual intercourse with many people can cause HPV infection, having sex before the age of 20 is a risk factor for cervical cancer, that use of condoms can prevent cervical cancer, cervical cancer screening with HPV DNA testing, HPV vaccine, that the HPV vaccine can prevent cervical cancer, the HPV vaccine can be given to 9-14-year-olds, and if they are willing to vaccinate their girl child.

The data was in the printed paper and entered and analysed using Microsoft Excel 2016. The point estimate was calculated at a 95% Confidence Interval.

## RESULTS

The questionnaires were distributed to more than 600 patients to adjust the non-respondents. A total of 583 patients returned the self-reported questionnaires. After removing missing data, a total of 508 questionnaires were analysed. Among them, the pooled knowledge of Cervical Cancer Screening and Prevention by Human Papillomavirus Deoxyribonucleic Acid and Human Papillomavirus Vaccination was found to be 42 (8.25%) (5.86-10.64, 95% Confidence Interval) from 11 questionnaires (Table 1).

Among 508 patients, 164 (32.28%) know about cervical cancer, 15 (2.95%) know about HPV infection, 14 (2.76%) know about HPV infection causes cervical cancer, 21 (4.13%) know about HPV transmitted through multiple sex partners, 29 (5.71%) awareness about young age sexual activity (less than 16 years) causing cervical cancer, 32 (6.30%) know about condom prevents cervical cancer, 15 (2.95%) knowledge on cervical ca screening and prevention by HPV DNA, 12 (2.36%) awareness on HPV vaccine, 21 (4.13%) HPV vaccine prevents cervical cancer, 37 (7.28%) awareness on HPV vaccine to 9-13 years female child, 101 (19.88%) willing to vaccinate female child (Table 1).

**Table 1:** Questionnaires on knowledge of cervical cancer screening and prevention by human papillomavirus deoxyribonucleic acid and human papillomavirus vaccination (n= 508).

Questionnaires	Yes, n (%)	No, n (%)
Do you know about cervical cancer?	164 (32.28)	344 (67.72)
Do you know about HPV?	15 (2.95)	493 (97.05)
Did you know that the HPV virus can cause cervical cancer?	14 (2.76)	494 (97.24)
Did you know that having sexual intercourse with many people can cause HPV infection?	21 (4.13)	487 (95.87)
Did you know that having sex before the age of 20 is a risk factor for cervical cancer?	29 (5.71)	479 (94.29)
Did you know that the use of condoms can prevent cervical cancer?	32 (6.30)	476 (93.70)
Do you know about cervical cancer screening with HPV DNA testing?	15 (2.95)	493 (97.05)
Do you know about the HPV vaccine?	12 (2.36)	496 (97.64)
Did you know that the HPV vaccine can prevent cervical cancer?	21 (4.13)	487 (95.87)
Did you know that the HPV vaccine can be given to 9-14-year-olds?		37 (7.28)	471 (92.72)
Are you ready to vaccinate your girl child?	101 (19.88)	407 (80.12)

**Figure 1:**
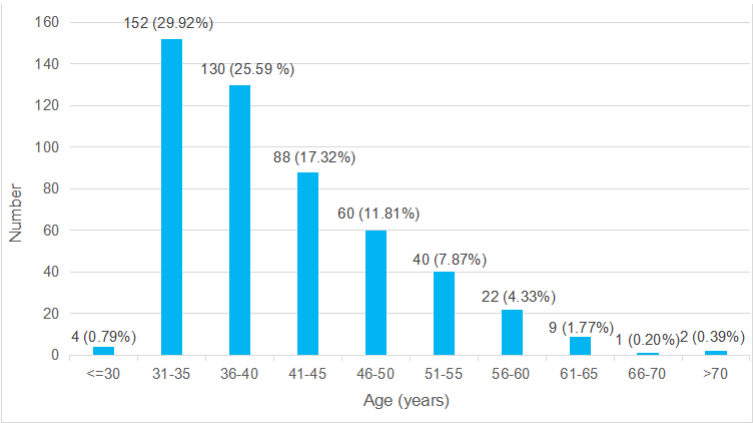
Age-wise distribution of the participants (n = 508).

In a total sample of 508 patients, 152 (29.92%) were under the age group 31-35 years whereas only 1 (0.20%) woman was of the age group 66-70 years (Figure 1).

**Figure 2:**
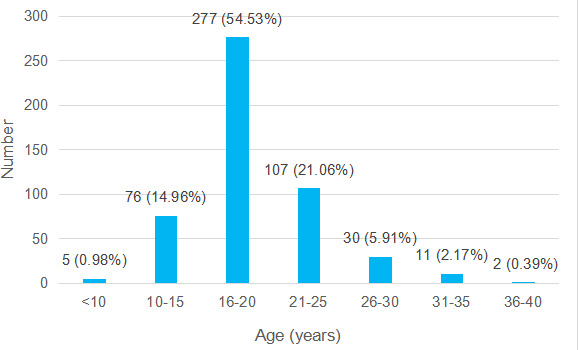
Age of first sexual intercourse among patients (n= 508).

Among total samples, 358 (70.47%) had their first sexual intercourse under the age of 20 followed by 107 (21.06%) among the age group of 21-25 (Figure 2). More than half the participants had their first sexual intercourse among the age group of 16-20.

Among 508 patients, 261 (51.38%) were illiterate followed by 61 (12.20%) had completed their education up to class 10. Three hundred and fifty-two (69.29%) were housewives by profession followed by 61 (12.01%) were farmers (Table 2).

**Table 2:** Educational and professional status of the patients (n= 508).

Educational status	n (%)
Up to class 5	45 (8.86)
Up to class 10	62 (12.20)
SLC/SEE	41 (8.07)
Plus 2	38 (7.48)
Bachelors plus	49 (9.65)
Illiterate	261 (51.38)
Profession	n (%)
Housewife	352 (69.29)
Agriculture	61 (12.01)
Business	22 (4.33)
Teacher	13 (2.56)
Others	60 (11.81)

## DISCUSSION

Among 508 women, 42 (8.25%) had knowledge of cervical cancer screening and prevention by human papillomavirus deoxyribonucleic acid and human papillomavirus vaccination.

In this study, out of 508 participants, only 164 (32.28%) had heard about cervical cancer. In a study done in past, (65.5%) had heard of cervical cancer.^6^ In a similar study, it was found that 90.6% were aware of cervical cancer.^7^ In another study, 85.11% were aware of cervical cancer.^8^ Regarding cervical cancer screening, in our study, 15 (2.95%) knew about the HPV DNA test. In a similar study, 83.3% were aware that PAP (Papanicolaou) smear test detects cervical cancer.^7^

In our study, only 14 (2.76%) knew that HPV infection can cause cervical cancer. In a similar study done in past, 86.2% of the respondents knew that HPV causes cervical cancer.^7^

In our study, 12 (2.36%) knew about the HPV vaccine whereas in a similar study, only about 13 (12.7%) had heard of a vaccine for cervical cancer. Only about 12 (2.36%) had heard of a vaccine for cervical cancer while about 496 (97.64%) had not heard of it. In a similar study, 29.2% of the eligible respondents underwent screening against cervical cancer, and 19.8% of the study participants were vaccinated for HPV.^7^

A majority of participants 487 (95.87%) were not aware that HPV infection is transmitted through multiple sexual partners. However, only 6.30% had knowledge about the prevention.^9^

Only 21.5% of the participants knew HPV as the cause of cervical cancer; 13.9% were aware of an HPV vaccine; and 96% reported that they would have their children vaccinated against HPV if the vaccine was available free of cost to them.^10^

Regarding the study on HPV vaccination, only a few of the participants (2.36%) knew about HPV vaccines. In a study, it was shown that 1.4% of the total female population received a full course of vaccines.11 While most 471 (92.72%) of the participants had no knowledge that the vaccine should be given at the age of 9-12 years. Only 101 (19.88%) were willing to vaccinate their child in our study while 407 (80.12%) are against vaccination. In a similar study, 77.2% of the respondents were willing to be vaccinated and recommend HPV vaccination to their family members.^7^

This study has several limitations. Since this is a descriptive cross-sectional study conducted in a single setting, the findings can not be generalized to a broader level. Also, due to the limitations of the study design, analytical parameters like association could not be established.

## CONCLUSIONS

The knowledge of cervical cancer screening and prevention by human papillomavirus deoxyribonucleic acid and human papillomavirus vaccination among women was found to be very low. Hence, appropriate education and counselling should be given through awareness programs to women from various national and international efforts.
